# Derby Sanatorium Inquiry

**Published:** 1920-08-14

**Authors:** 


### Derby Sanatorium Inquiry.

### THE ALLEGATIONS AND REPLIES.

An inquiry has been held into the treatment of ex-soldiers at the Derby Sanatorium.

The men, who brought forward eighteen witnesses, alleged that the food was badly kept, cooked, and served, and thus "transformed" from good to bad quality. Herrings were served without having been cleaned. When the patients had thrown some of these out of the window they had been told that the same food would be served to them again one or two days hence. There were maggots in the meat, and the milk, which was dirty, was served in vessels used for washing potatoes. Sometimes the food was raw. The time between each course was often half an hour, and billiards was played to beguile the interval. Fish skins and mice dirt were fo[illegible] in the porridge.

On behalf of the Health Committee, the Town C[illegible] replied that it was noteworthy that the women, who [illegible] the same food as the men, made no complaints. [illegible] number of instances at the root of the complaints pro[illegible] on investigation to be very few. Once a piece was fo[illegible] in the porridge.

Dr. Brindley, against whom and his medical collea[illegible] no complaint had been made, said that he had freque[illegible] inspected the food and always found it satisfactory. [illegible] R. B. Berry confirmed this statement, as did Miss Be[illegible] the Matron, who was closely examined by the T[illegible] Clerk. These statements closed the inquiry.

